# Hydration Assessment in Males and Females Using the WUT (Weight, Urine Color, and Thirst) Venn Diagram Compared to Blood and Urinary Indices

**DOI:** 10.3390/nu17040689

**Published:** 2025-02-14

**Authors:** Marcos S. Keefe, Hui-Ying Luk, Jan-Joseph Rolloque, Nigel C. Jiwan, Yasuki Sekiguchi

**Affiliations:** 1 Sports Performance Laboratory, Department of Kinesiology and Sport Management, Texas Tech University, Lubbock, TX 79409, USA; makeefe@ttu.edu (M.S.K.); jarolloq@ttu.edu (J.-J.R.); 2 Applied Physiology Laboratory, Department of Kinesiology and Sport Management, Texas Tech University, Lubbock, TX 79409, USA; huiying.luk@ttu.edu (H.-Y.L.); jiwan@hope.edu (N.C.J.); 3 Department of Kinesiology, Hope College, Holland, MI 49423, USA

**Keywords:** dehydration, sex differences, field settings, Venn diagram, hydration assessment

## Abstract

**Objectives**: The WUT (weight, urine color, thirst) Venn diagram has been demonstrated as a practical tool for hydration assessment. However, no investigations have examined if there are sex differences in the accuracy of this method. Therefore, the purpose of this study was to explore potential sex differences in the accuracy of the WUT Venn diagram for hydration status determination. **Methods**: Twelve men (21 ± 2 years; 81.0 ± 15.9 kg) and twelve women (22 ± 3 years; 68.8 ± 15.2 kg) completed the study. Body mass, urine color, urine specific gravity (USG), urine osmolality (UOSM), thirst level, and plasma osmolality (POSM) were collected at first-morning and afternoon visits for three consecutive days in free-living and euhydrated states. The number of markers indicating dehydration levels were categorized into either 3, 2, 1, or 0 WUT markers. Receiver-operating characteristics analysis calculated the sensitivity and specificity of 1, 2, or 3 hydration markers in detecting dehydration or euhydration. **Results**: WUT2 and WUT3 resulted in high specificity values in comparison to USG (WUT2—male: 0.912, female: 0.949; WUT3—male: 0.991, female: 1.000), UOSM (WUT2—male: 0.901, female: 0.946; WUT3—male: 0.991, female: 1.000), and POSM (WUT2—male: 0.859, female: 0.896 WUT3; male: 0.977, female: 0.991). WUT1 resulted in high sensitivity values compared to USG (male: 0.931, female: 0.857), but varied between sexes for UOSM (male: 0.875, female: 0.794) and POSM (male: 0.469, female:0.536). **Conclusions**: The WUT Venn diagram accurately detects dehydration when two or three WUT markers are met in both males and females. The WUT Venn diagram accurately assesses hydration status in males and females, thus providing medical personnel and athlete support teams a cost-effective and time-efficient hydration testing tool.

## 1. Introduction

Hydration is an essential component for a variety of physiological factors related to health and exercise performance [1,2]. For example, a euhydrated state (“normal” body water content) may attenuate rises in thermal, physiological, and perceptual strain during exercise [3,4,5]. However, assessment of hydration status remains a controversial topic within the scientific community, especially during assessment of athletic, military, and occupational populations in field settings [6,7,8].

Current hydration literature is largely comprised of male participants which results in unclear transferable conclusions to the female population [2]. One of the main factors that is thought to influence sex differences in hydration is the role of sex hormones (e.g., estrogen and progesterone) and their impact on other volume regulatory hormones, such as arginine vasopressin [4,9]. In addition, absolute and relative total body water are lower in females compared with males due to the typical lower total body mass and smaller stature of females [10]. The prevalence of studying sex differences is highlighted by the increasing participation of women in sport and exercise, military, and occupational settings [2,11]. Therefore, additional research needs to emerge within the hydration field to ensure proper assessment of all individuals.

Numerous methodologies for hydration assessment exist, including blood, urine, body mass, and perceptual indices; however, many require high technique complexity and equipment cost, or are invasive and require venous blood draws [6,12]. Altogether, several of these techniques are not applicable to implement in field settings where individuals require immediate feedback in a cost-efficient manner. Consequently, a Venn diagram decision tool comprised of three simple hydration markers (weight [body mass loss], urine color, and thirst [WUT]) was created to practically assess hydration status in field settings, specifically athletic settings [8]. When two of the three markers meet the dehydration threshold for each marker, then the individual is ‘likely dehydrated’, and when all three markers are met then the individual is ‘very likely dehydrated’ [8]. The use of this hydration tool has been shown to be an accurate predictor of hydration status [13,14]; however, it remains unclear whether this accuracy varies between males and females.

Given the paucity of female inclusion in hydration research, there is a need to further understand if sex differences exist with a practically relevant hydration assessment tool in field settings. Although physiological factors suggest there are sex differences in hydration status, a hydration assessment tool should not be influenced by these differences and needs to accurately assess hydration independent of sex. Thus, this study aimed to explore sex differences in the accuracy of the WUT Venn diagram in determining hydration status compared to blood and urine hydration indices. It is hypothesized that the WUT Venn diagram would result in high accuracy for males and females when determining dehydration by meeting two or three WUT markers.

## 2. Materials and Methods

### 2.1. Participants

The present research is part of a larger study and thus includes the same data set of participants [13]. This study was approved by the Texas Tech University Institutional Review Board (2022-640) for human subject research and adhered to the Declaration of Helsinki. Written informed consent and a medical screening questionnaire were obtained from each participant before participation in the study. A total of twenty-four participants, twelve men (mean ± SD; age: 21 ± 2 years; mass: 81.0 ± 15.9 kg) and twelve women (age: 22 ± 3 years; mass: 68.8 ± 15.2 kg), volunteered to participate in this study. A power analysis conducted with G*Power 3.1.9.7 (Universitat Kiel, Kiel, Germany) determined that twenty-four participants were needed in the present study for a power of 0.80, with an effect size of 0.2 and an alpha level of 0.05 [14]. All participants reported not having kidney disease or a urinary tract infection at the time of the study. In an attempt to control for menstrual cycles, only women using an oral contraceptive pill (OCP) were recruited to participate in this study and completed the study visits during the 7-day placebo pill time frame, which is designed to occur during the menstruation phase [15].

### 2.2. Experimental Procedures

Participants visited the laboratory on twelve occasions across a seven-day time frame. Visits were performed in the morning and afternoon of three consecutive days under free-living (FL) conditions. Researchers instructed participants to maintain their habitual lifestyle during the FL conditions, including eating, drinking, and exercising. Following a one-day break, the remaining visits were performed in the morning and afternoon for three consecutive days in a euhydrated (EUH) state. Euhydration was defined as providing a spot urine sample with a urine specific gravity (USG) <1.020. Researchers provided participants with fluid intake reminders every 3 h throughout the day to ensure participants would be EUH during these visits. Table 1 demonstrates hydration state according to the hydration variables during the FL and EUH conditions (average across the three days for each condition). All morning visits were performed as a first-morning spot sample. Participants were instructed to arrive at the laboratory having abstained from any food or fluid consumption, or exercise. Urine cups were provided to participants the day prior so that the first-morning urine sample could be provided to researchers upon visitation to the laboratory. In addition, a venous blood sample, body mass (BM), and thirst level were collected at each visit. All afternoon visits were performed between 2:00 and 4:00 p.m. and followed the same procedures as the morning visits.

### 2.3. Measurements

Urine indices (USG, urine osmolality [UOSM], and urine color [UCOL]), hematologic indices (plasma osmolality [POSM]), BM, and thirst level were collected at each visit. USG was measured using a handheld refractometer (ATAGO, Tokyo, Japan) and UCOL was assessed via a validated 8-point UCOL chart [16]. UOSM and POSM were analyzed via an Advanced Instruments Osmometer Pro (Norwood, MA, USA), with each sample being measured in duplicate.

Nude BM was measured via an electronic scale (Health-o-Meter). Percentage body mass loss (BML) for each day was calculated based on the average of the three euhydrated morning BM measurements for each participant: ([BM of each day − Baseline BM] × Baseline BM-1 × 100). Thirst level was assessed on a Likert-type scale of 1 to 9, with 1 being “not thirsty at all” and 9 being “very, very thirsty” [17].

### 2.4. WUT Criteria Determination

Dehydration thresholds were previously determined for the three WUT markers and if any of these criterions were met, then a score of “1” was aggregated towards the final count. The total number of markers that indicated dehydration were counted and categorized into either 0, 1, 2, or 3 WUT markers (WUT0, WUT1, WUT2, and WUT3) and were compared to hematologic and urinary indices. A BML >1%, UCOL ≥ 5, and thirst level ≥5 were the designated dehydration thresholds [14,18]. In comparison to hematologic and urinary hydration markers, a USG ≥1.020, UOSM >700 mOsmol, and POSM >290 mOsmol indicate dehydration based on standards of the American College of Sports Medicine (ACSM) [1].

### 2.5. Statistical Analyses

All statistical analyses were performed using SPSS Statistics software (Version 25; IBM Corporation, Armonk, NY, USA). Data are presented as mean ± SD, with an alpha level of *p* ≤ 0.05 demonstrating significance for all comparisons. One-way ANOVA with Tukey pairwise comparisons was used to assess differences in USG, UOSM, and POSM for the different numbers of WUT markers between sexes as each urine and blood sample was treated as an individual sample. Receiver-operating characteristics (ROC) analysis (i.e., sensitivity and specificity) was performed to calculate the predictive value of 0, 1, 2, or 3 hydration markers in detecting a dehydrated or euhydrated state, which were defined by USG, UOSM, and POSM. Cutoff determination values were calculated based off the calculated sensitivity and specificity values [19]. Positive and negative predictive values provide additional context for the WUT indices to accurately predict hydration state according to USG, UOSM, and POSM. High sensitivity corresponds to the WUT Venn diagram being accurate in determining euhydration, whereas high specificity corresponds with it accurately determining dehydration.

## 3. Results

**Figure 1:**
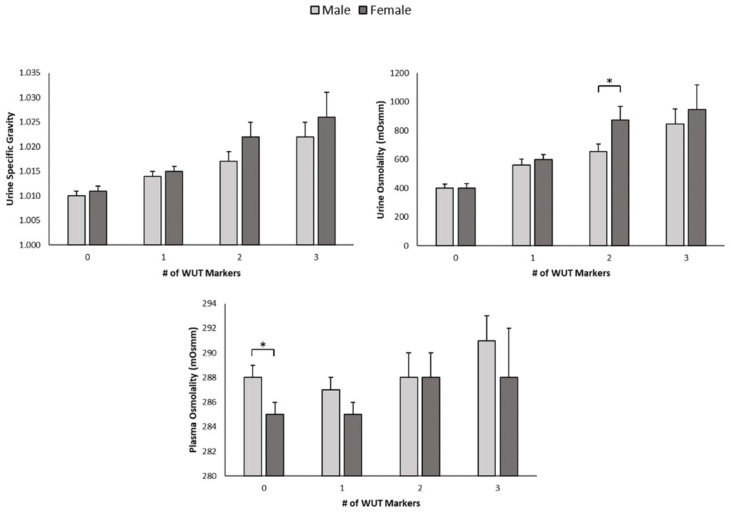
Male and female urine specific gravity, urine osmolality, and plasma osmolality when weight, urine color, and thirst (WUT) Venn diagram criteria were used to determine hydration status. * Indicates significant difference between sexes (*p* < 0.05).

A total of 288 samples were analyzed for USG and UOSM, and 271 samples for POSM. A total of 17 plasma samples were missed because of technical issues. Of the 17 missed plasma samples, 8 of these were from male participants and 9 from female participants. Mean values of USG, UOSM, and POSM for each WUT category for males and females are presented in Figure 1.

### Receiver-Operating Characteristics for Males and Females

**Figure 2:**
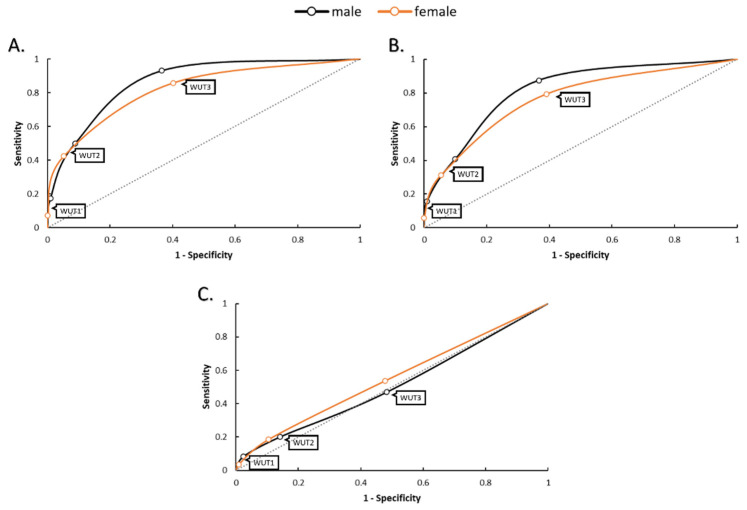
Receiver-operating characteristic (ROC) curves for males and females for (**A**) urine specific gravity, (**B**) urine osmolality, and (**C**) plasma osmolality. WUT1, WUT2, and WUT3 thresholds are plotted appropriately.

Figure 2 presents ROC curves of sensitivity and specificity values for males and females when WUT criteria were used to determine hydration status in comparison to USG, UOSM, and POSM. In both males and females, WUT2 and WUT3 resulted in high specificity values in comparison to USG (specificity = 0.912–1.000), UOSM (specificity = 0.901–1.000), and POSM (specificity = 0.859–0.991). In males, WUT1 resulted in high sensitivity values compared to USG (sensitivity = 0.931) and UOSM (sensitivity = 0.875), but not POSM (sensitivity = 0.469). In females, WUT1 resulted in high sensitivity values compared to USG (sensitivity = 0.857), but not UOSM (sensitivity = 0.794) or POSM (sensitivity = 0.536).

Table 2 and Table 3 present sensitivity and specificity values for males and females, respectively, when investigating EUH and FL conditions separately. In both males and females, WUT3 resulted in high specificity values in comparison to USG (specificity = 0.977–1.000), UOSM (specificity = 0.977–1.000), and POSM (specificity = 0.952–1.000) for both EUH and FL conditions. Similarly, WUT2 resulted in high specificity values in males and females for USG (specificity = 0.841–0.972) and UOSM (specificity = 0.814–0.971) for EUH and FL, but not for FL males for POSM (specificity = 0.738). WUT1 resulted in high sensitivity values for males and females for the FL condition in comparison to USG (sensitivity = 0.929), UOSM (sensitivity = 0.813–0.931), and only female POSM (sensitivity = 0.917), but not male POSM (sensitivity = 0.731). However, WUT1 in the EUH condition did not result in a high sensitivity for either sex for UOSM (sensitivity = 0.333–0.500) or POSM (sensitivity = 0.174–0.250) and was not applicable for USG.

## 4. Discussion

This investigation was to examine the accuracy of the WUT Venn diagram compared to blood and urinary indices for hydration status assessment between sexes. Results of the present study revealed that both males and females demonstrated high accuracy for determination of dehydration when 2 or 3 WUT markers were met in comparison to USG, UOSM, and POSM. In addition, meeting 1 WUT marker did not result in an accurate determination of dehydration, which is in lieu of the original structure of the WUT Venn diagram [8]. Furthermore, analyzing the accuracy between conditions (EUH vs. FL) resulted in similar WUT2 and WUT3 (high specificity values) results in both sexes, aside from FL male when compared to POSM. These findings highlight that the WUT Venn diagram is an accurate hydration testing tool for males and females, thus expanding its application in settings involving both sexes.

Hydration literature has historically been investigated solely in males, neglecting female investigation, and has thus resulted in a lack of appropriate findings for females [2]. Recently, Adams et al. (2020) explored potential sex differences in 24 h urinary hydration markers (UCOL, USG, UOSM, and urine volume) in young adults and found no significant differences in any of the hydration markers [20]. This study is not in lieu of previous studies which show that men generally have higher urinary hydration markers compared to women during rest, daily activities, and exercise or sport [21,22,23]. These discrepancies may be explained by the menstrual cycle, specifically the phase that female participants were currently undergoing during their participation in these studies, which was not recorded or measured via reproductive hormones. Although the current investigation focused on the accuracy of the WUT Venn diagram for males and females individually rather than a comparison of hydration status between sexes, our results demonstrate that this assessment tool is accurate in detecting dehydration in both males and females. Thus, even if sex differences inherently exist between urinary hydration markers, the WUT Venn diagram can accurately assess hydration status in both sexes.

In this study, USG, UOSM, and POSM were used as the external variables for the determination of hydration status in comparison to the WUT Venn diagram. Two of the most common methods for measuring hydration status include USG and UOSM, where previous research has demonstrated that these two variables are typically linearly correlated and can be used interchangeably [12,24,25]. While urinary indices are highly field-applicable for most scenarios, these measurements can be altered with rapid fluid intake, thus reducing the reliability of measurement [26]. Hematologic indices are typically considered more precise and accurate in hydration assessment [1], with POSM being the main hematologic variable assessed for hydration status determination. All three of these indices demonstrated high specificity values when being compared to the WUT Venn diagram in males and females; however, POSM resulted in lower specificity values (0.859 and 0.896) when 2 WUT markers were met compared to USG (0.912 and 0.949) and UOSM (0.901 and 0.946). Still, the POSM specificity values were higher than the cutoff determination values (0.660 and 0.675), allowing for the interpretation that meeting two WUT markers accurately determines dehydration.

Although the WUT Venn diagram was first proposed as a hydration assessment method in 2005 [8], there are very few original research studies that have investigated its accuracy in hydration assessment compared to other commonly used hydration indices [13,14,27,28]. Sekiguchi et al. (2022) [13] suggested that when all three WUT markers were met, urinary indices of USG and UOSM indicated dehydration, whereas meeting two WUT markers was not a strong enough indicator in college-aged individuals. Similarly, Wardenaar and colleagues (2023) [27] confirmed that meeting all three WUT markers suggested a USG above the 1.020 cut-off in a group of Army Reserve Officer Training Corps cadets and an individual from a club sports program at the university. Adams et al. (2024) [28] concluded that all three WUT markers could be used in conjunction, as well as independently, to determine the adequacy of fluid intake on a day-to-day basis from first-morning timepoints during both free-living and fluid-balance manipulation protocols. Although all three of these studies included female participants (n = 22 from Sekiguchi et al. (2022) [13]; n = 6 from Wardenaar et al. (2023) [27]; n = 47 from Adams et al. (2024) [28]), no study assessed potential sex differences in the accuracy of the WUT Venn diagram, and only Adams et al. (2024) [28] disclosed if they controlled for the menstrual cycle during data collection (testing performed during early follicular phase). As part of the same study, our group also determined that meeting three WUT markers resulted in high accuracy compared to urinary (USG and UOSM) and hematologic (POSM) indices at various timepoints throughout the day in both euhydrated (defined as USG <1.020) and free-living conditions [13]. The current findings further demonstrate that males and females likewise result in high accuracy of dehydration determination when two or three WUT markers are met compared to USG, UOSM, and POSM. The lack of disparities between sexes is logical as only females on OCPs participated in the study during their placebo-pill week. Indeed, the placebo-pill week is designed to occur during the menstruation phase while reproductive hormones that may affect fluid balance are at their lowest concentrations [2].

Three limitations are acknowledged by the researchers for this study. First, one variable of the WUT Venn diagram is a urinary measure (UCOL) which is directly related to two hydration variables (USG and UOSM), which were compared to the WUT Venn diagram. A third hydration variable (POSM) was included in the study as an external validating factor. Second, this study did not control BM for the visit collection at the afternoon timepoint. Food and fluid consumption, along with exercise, are factors that are likely to influence an individual’s BM. Although limiting, this factor was not controlled in order to maintain the FL condition and determine the accuracy of the WUT Venn diagram in a true FL situation. Additionally, the current dataset included both morning and afternoon samples, and our previous work demonstrates that these findings apply to both morning and afternoon timepoints [13]. Third, although the accuracy of the WUT Venn diagram was assessed in females, only female participants who were actively taking OCPs were recruited for this study. In addition, female participation occurred during the placebo-pill week to mimic the menstruation phase where levels of estrogen and progesterone are at their lowest. This was planned to limit the effect of hormonal effect on fluid balance, yet results in being unable to adapt the present investigation’s findings to females across the entire menstrual cycle. Further research should be conducted during each phase of the menstrual cycle to investigate whether the WUT Venn diagram can still accurately detect hydration status during varying levels of hormonal fluctuations and subsequent fluid balance changes.

## 5. Conclusions

The WUT Venn diagram accurately detects dehydration when two or three WUT markers are met in both males and females. However, when only one WUT marker is met, neither dehydration nor euhydration can be determined for either sex. Findings from this investigation allow for greater transferability of a practical hydration assessment tool not only for males but also for females in field settings where a cost-effective and time-efficient procedure is required. Athlete support teams, military personnel, field occupational workers, or individuals in field settings can use the WUT Venn diagram to determine the status of hydration in males and females.

## Figures and Tables

**Table 1:** Hydration marker (body mass [BM], BM loss [BML], urine color [U_COL_], urine specific gravity [USG], urine osmolality [UOSM], and plasma osmolality [POSM]) descriptive values of males and females for both euhydrated (EUH) and free-living (FL) conditions. Values are presented as mean ± SD.

	EUH Males	FL Males	EUH Females	FL Females
BM (kg)	81.33 ± 15.05	81.00 ± 15.04	69.07 ± 14.77	69.04 ± 14.68
BML (%)	−0.2 ± 0.7	0.2 ± 1.1	−0.3 ± 0.7	−0.2 ± 1.0
U_COL_	3 ± 1	4 ± 2	3 ± 2	4 ± 2
Thirst	3 ± 2	4 ± 2	3 ± 2	4 ± 2
USG	1.009 ± 0.005	1.016 ± 0.008	1.009 ± 0.005	1.017 ± 0.009
UOSM (mmol·kg^−1^)	376 ± 187	619 ± 268	364 ± 189	660 ± 307
POSM (mmol·kg^−1^)	287 ± 5	289 ± 5	285 ± 6	285 ± 5

**Table 2:** Male participants’ sensitivity, specificity, cutoff determination value, positive predictive value (PPV), and negative predictive value (NPV) for euhydrated (EUH) and free-living (FL) conditions urine specific gravity (USG), urine osmolality (UOSM), and plasma osmolality (POSM) when weight, urine color, and thirst (WUT) Venn diagram criteria were used to determine hydration status (USG > 1.020, UOSM > 700 mOsm, and POSM > 290 mOsm).

	# of WUT Markers	Sensitivity	Specificity	Cutoff Determination Value	PPV (%)	NPV (%)
		EUH: FL				
USG	3	0.000: 0.179	1.000 *: 0.977 *	NA: 0.675	NA: 83.3	98.6: 65.2
	2	0.000: 0.607	0.944 *: 0.841 *	NA: 0.180	0: 70.8	98.5: 77.1
	1	1.000 *: 0.929 *	0.732: 0.477	0.072: 0.279	5.0: 53.1	100.0: 91.3
UOSM	3	0.000: 0.172	1.000 *: 0.977 *	NA: 0.686	NA: 83.3	95.8: 63.6
	2	0.000: 0.552	0.942 *: 0.814 *	NA: 0.235	0: 66.7	95.6: 72.9
	1	0.333: 0.931 *	0.725: 0.488	0.521: 0.267	5.0: 55.1	96.2: 91.3
POSM	3	0.000: 0.154	1.000 *: 0.952 *	NA: 0.718	NA: 66.7	66.2: 64.5
	2	0.044: 0.462	0.933 *: 0.738	0.918: 0.358	25.0: 52.2	65.6: 68.9
	1	0.174: 0.731	0.689: 0.333	0.779: 0.517	22.2: 40.4	62.0: 66.7

* Indicates sensitivity/specificity > cutoff determination value and >0.800, which determines euhydration vs. dehydration. NA: not applicable.

**Table 3:** Female participants’ sensitivity, specificity, cutoff determination value, positive predictive value (PPV), and negative predictive value (NPV) for euhydrated (EUH) and free-living (FL) conditions urine specific gravity (USG), urine osmolality (UOSM), and plasma osmolality (POSM) when weight, urine color, and thirst (WUT) Venn diagram criteria were used to determine hydration status (USG > 1.020, UOSM > 700 mOsm, and POSM > 290 mOsm).

	# of WUT Markers	Sensitivity	Specificity	Cutoff Determination Value	PPV (%)	NPV (%)
		EUH: FL				
USG	3	NA: 0.179	1.000 *: 0.977 *	NA: 0.675	NA: 83.3	100.0: 65.2
	2	NA: 0.607	0.972 *: 0.841 *	NA: 0.180	0: 70.8	100.0: 77.1
	1	NA: 0.929 *	0.694: 0.477	NA: 0.279	0: 53.1	100.0: 91.3
UOSM	3	0.000: 0.063	1.000 *: 1.000 *	NA: NA	NA: 100.0	97.2: 57.1
	2	0.000: 0.375	0.971 *: 0.900 *	NA: 0.401	0: 75.0	97.1: 64.3
	1	0.500: 0.813 *	0.700: 0.450	0.340: 0.337	4.5: 54.2	98.0: 75.0
POSM	3	0.000: 0.083	1.000 *: 0.983 *	NA: 0.841	-: 50.0	75.8: 83.6
	2	0.000: 0.500	0.960 *: 0.825 *	NA: 0.281	0: 37.5	75.0: 88.7
	1	0.250: 0.917 *	0.700: 0.368	0.653: 0.406	21.1: 23.4	74.5: 95.5

* Indicates sensitivity/specificity > cutoff determination value and >0.800, which determines euhydration vs. dehydration. NA: not applicable.

## Data Availability

The data presented in this study are available on request from the corresponding author.
